# A *brittle‐2* transgene increases maize yield by acting in maternal tissues to increase seed number

**DOI:** 10.1002/pld3.29

**Published:** 2017-12-07

**Authors:** L. Curtis Hannah, Janine R. Shaw, Maureen A. Clancy, Nikolaos Georgelis, Susan K. Boehlein

**Affiliations:** ^1^ Program in Plant Molecular and Cellular Biology Department of Horticultural Sciences University of Florida Gainesville FL USA; ^2^ Present address: Simplot Plant Sciences J.R. Simplot Company Boise ID USA

**Keywords:** abiotic plant stress, ADP‐glucose pyrophosphorylase, maize yield, starch synthesis

## Abstract

The enzyme ADP‐glucose pyrophosphorylase is essential for starch biosynthesis and is highly regulated. Here, mutations that increased heat stability and interactions with allosteric effectors were incorporated into the small subunit of the isoform known to be expressed at high levels in the maize endosperm. The resulting variants were transformed into maize with expression targeted to the endosperm. Transgenes harboring the changes increased yield some 35%; however, yield enhancement occurred via an increase in seed number rather than by increased seed weight. Interestingly, seed number increase is controlled by the genotype of the plant rather than the genotype of the seed as seeds increase in number whether or not they contain the transgene as long as the maternal parent has the transgene. The transgene is however expressed in the endosperm, and the altered allosteric and stability properties initially seen in *Escherichia coli* expression experiments are also seen with the endosperm‐expressed gene. The extent of seed number increase is positively correlated with the average daily high temperature during the first 4 days postpollination. While these results were unexpected, they echo the phenotypic changes caused by the insertion of an altered large subunit of this enzyme reported previously (*Plant Cell*,* 24*, 2012, 2352). These results call into question some of the reported fundamental differences separating starch synthesis in the endosperm vis‐à‐vis other plant tissues.

## 1 INTRODUCTION

Starch is the major component of cereal seeds and hence a critical constituent of cereal seed yields. The importance of starch, coupled with the early availability of seminal mutants in important biosynthetic steps, has allowed for a fairly robust understanding of its biochemical pathway (reviewed in Smith, Denyer, & Martin, 1997; Ballicora, Iglesias, & Preiss, 2003; Hannah, 2007; Hannah & Greene, 2008; Hannah & James, 2008; Preiss, 2009; Keeling & Myers, 2010).

A series of studies have pointed to the enzyme, ADP‐glucose pyrophosphorylase (AGPase) as a key step in this important pathway. AGPase is composed of two identical small and two identical large subunits and synthesizes ADP‐glucose from glucose‐1‐PO_4_ and ATP. The glucose of ADP‐glucose is then used to extend the growing chain of the polysaccharides, amylose and amylopectin. AGPase, the first step unique to the starch biosynthetic pathway, is allosterically regulated and the isoform in the cereal endosperm is heat‐labile (reviewed in Hannah, 2007). Results from various laboratories show that incorporation of an altered AGPase can enhance starch production. Expression of altered AGPases producing increased yield has been reported in potato (Stark, Timmerman, Barry, Preiss, & Kishore, 1992), lettuce (Lee, Ryu, Kim, Okita, & Kim, 2009), maize (Giroux et al., 1996; Hannah et al., 2012; Li, Zhang, Zhao, Li, & Zhang, 2011; Wang et al., 2007), wheat (Smidansky et al., 2002), rice (Sakulsingharoj et al., 2004 and Smidansky, Martin, Hannah, Fischer, & Giroux, 2003), and Arabidopsis (Obana et al., 2006).

Because of the proposed importance of the allosteric and heat stability characteristics of AGPase, we used direct selection in a bacterial system as well as evolutionary and structural considerations to pinpoint and alter amino acids in both subunits of the maize endosperm AGPase that control these two characteristics of the enzyme (Boehlein, Shaw, Georgelis, & Hannah, 2014; Boehlein, Shaw, Stewart, Sullivan, & Hannah, 2015; Cross et al., 2004; Georgelis & Hannah, 2008; Georgelis, Shaw, & Hannah, 2009; Giroux et al., 1996; Greene and Hannah, 1998a,b; Hannah et al., 2012; Linebarger, Boehlein, Sewell, Shaw, & Hannah, 2005). Here, we focus on the field performance of two of these variants in the small subunit termed MP and MP‐QTCL.

The small and large subunits of the maize endosperm AGPase are encoded by the genes *brittle‐2* (*bt2*) and *shrunken‐2* (*sh2*), respectively. Previously, we showed that a chimeric small subunit in which the N‐terminal 198 amino acids were derived from the BT2 protein and the carboxyl‐terminal 277 amino acids originated from the potato tuber small subunit (termed MP) polymerized with the SH2 protein to produce an enzyme in an *Escherichia coli* expression system that exhibited a *k*_cat_ some 10‐fold greater than the wildtype maize endosperm in the absence of the activator, 3‐phosphoglyceric acid (3PGA). In addition, the MP small subunit conditioned a more heat‐stable AGPase compared to the wildtype maize endosperm enzyme (Boehlein, Sewell, Cross, Stewart, & Hannah, 2005; Boehlein, Shaw, Stewart, & Hannah, 2008, 2009; Cross et al., 2005). Significantly, while 3PGA activates both the maize endosperm and potato tuber enzymes, the mechanisms of activation are fundamentally different. Whereas the activator enhances substrate binding of the maize enzyme, it enhances product release of the potato enzyme. Interestingly, the MP‐encoded AGPase still exhibits the 3PGA activation kinetics of the maize enzyme (Boehlein, Shaw, Hwang, Stewart, & Hannah, 2013). In addition, while both the potato tuber and the maize endosperm AGPases are inhibited by phosphate, the two enzymes exhibit markedly different mechanisms of inhibition. The mechanism of phosphate inhibition of the MP AGPase closely resembles that of the potato enzyme (Boehlein, Shaw, McCarty et al., 2013).

Sequence comparisons of heat‐stable and heat‐labile AGPase and subsequent mutagenesis experiments identified a cysteine in the N‐terminus of the small subunit that is involved in disulfide bridge formation (reviewed in Linebarger et al., 2005). The cysteine is also critical for heat stability. Placement of the cysteine‐containing motif QTCL into the N‐terminus of the chimeric MP small subunit enhanced heat stability more than 300‐fold at 58°C in comparison with the wildtype maize endosperm enzyme (Boehlein et al., 2005; Linebarger et al., 2005). This alteration also doubled the *k*_cat_ of the enzyme, enhanced sensitivity to the activator 3PGA but did not affect *K*_m_s for the physiological substrates, ATP and glucose‐1‐phosphate or the *K*_i_ of phosphate.

Here, we show that placement of MP and MP‐QTCL into maize enhances seed yield. However, the genes function in maternal tissue to enhance seed set. The extent of enhancement is proportional to daily high temperatures during the time of seed set. While the transgenes also function in the endosperm to complement the mutant phenotype, their function does not enhance seed weight. Characterization of AGPase from maize kernels expressing the MP small subunit showed that the plant‐derived enzyme has the altered properties first identified in the *E. coli* expression system. The unexpected finding of function in maternal tissue to increase seed number in a temperature‐dependent manner also has been found with an altered large subunit of the maize endosperm AGPase (Hannah et al., 2012).

## 2 MATERIALS AND METHODS

### 2.1 Plant material

Three AGPase small subunits were transgenically expressed in maize; the wildtype *Bt2* gene, the maize/potato (MP) chimeric gene and the MP gene containing the QTCL insertion (Cross et al., 2005; Linebarger et al., 2005). Coding sequences were cloned between the promoter and terminator of the maize gamma‐zein gene, ZmGZein27 obtained from the 6358 bp clone, pDAB 1450 (Ueda, Wang, Pham, & Messing, 1994). Resulting genes were placed into the Agrobacterium binary vector, pTF101 that contains the *bar* gene for Basta resistance. Following maize transformation into Hi‐II at the Iowa State University Plant Transformation Facility, regenerated plants were crossed with a stock mutant for the *Bt2* gene.

All plant materials were grown at the Plant Science Research and Education Center in Citra Florida. Plant culture involved the use of a starter fertilizer (18 kg/ha nitrogen and 63 kg/ha phosphate) at planting, and three side dressings during development with each regime involving 84 kg/ha of both N and P as well as micronutrients. Plants were irrigated twice per week if needed. Insecticides were sprayed twice/week and fungicides were used when needed. Fields for maize research are used each third season. Legumes are grown in these fields two of the three growing seasons. Plantings were staggered in seven‐day intervals over the spring growing season starting about April 15 of each year. Data were collected over a three‐year period.

Transgenic events that could complement the *bt2* mutation in the endosperm were propagated by selfing or crossing to the inbred B73 and were analyzed in several growing seasons. Complementation of the *bt2* mutation was evidenced by a 15 plump: 1 shrunken seed ratio in progeny derived by self‐pollination of the F1 generation. The presence of the transgene was monitored by painting a small portion of one leaf with Basta 7 to 10 days postpollination and scoring for resistance 7 days later. Selected transgenic events were also monitored for cosegregation of mutant seed with herbicide sensitivity. All materials for analysis were derived by self‐pollination performed after the individual plant shed pollen for 2 days.

The various transgenes were examined in three distinct genetic backgrounds. In one case, F_2_ seeds were planted, resulting plants were self‐pollinated, and seed number and individual weights of plump and mutant seed on each ear were determined. The presence of the transgene in each plant was monitored by Basta painting as described above. In other families, F_3_ seed from mixtures of families from sib F_2_ plants was planted and seed number and weight were monitored. Finally, seed derived from crossing Basta‐resistant F2 plants to the inbred B73 was planted. The source of the seed is listed in Table S1.

Temperature data were obtained through the Florida Automated Weather Network (https://fawn.ifas.ufl.edu/) for the Plant Science Research and Education Center in Citra, Florida.

### 2.2 Enzyme characterization

Developing kernels harvested 18 days postpollination were frozen in the field with liquid nitrogen, and shelled kernels were stored at −80°C. Frozen endosperms were removed from the embryo and pericarp and AGPase was purified by use of protamine sulfate and ammonium sulfate fractionation and ion exchange and hydroxyapatite chromatography as described previously (Boehlein et al., 2005). Purified AGPase was stored in the presence of 300 mM phosphate at −80°C. Before assay, preparations were desalted and immediately placed into a 1 mg/ml of bovine serum albumin (BSA) solution.

Catalytic stability of the AGPases was carried out in the presence of 50 mM HEPES pH 7.4, 15 mM MgCl_2_, 5 mM ATP, 2 mM G‐1‐P, and 2.5 mM 3PGA. Reactions proceeded for 2.5, 5, 10, or 15 min and were terminated by boiling. Activities were determined by linear portions of the curve.

The half‐life for each preparation was determined by incubating the enzyme in BSA at either 37°C or 55°C for 0, 1, 2.5, 5, 7.5, 10, 12.5, 15, 20, or 30 min. Following treatment, preparations were returned to ice. Controls remained on ice the entire time. Following heat treatment, activity at 37°C was determined in the reaction mixture described above. All reactions proceeded for 10 min and were terminated by boiling. The half‐time of inactivation (*t*_1/2_) was calculated from logarithmic plots of % activity versus time whereby the slope was equal to −*k*/(2.3). *T*_1/2_ was calculated from the equation *k *=* *0.693/*t*_1/2_. Activity in all experiments is reported in μmol/min/mg protein. Kinetic and allosteric parameters were determined by methods used previously (e.g., Boehlein et al., 2014, 2015).

## 3 RESULTS

### 3.1 The MP and MP‐QTCL AGPase genes increase yield by increasing seed number

Constructs containing the wildtype *Bt2* gene, the mosaic MP small subunit, or the MP subunit containing the QTCL motif as well as the *bar* gene for Basta resistance were synthesized and then transformed into maize by the Iowa State Maize Transformation Core. Regenerated plants were crossed with a stock containing a recessive, loss‐of‐function allele of the *bt2* gene.

Resulting seeds were planted at the Plant Science Research and Education Center in Citra Florida. Plants were self‐pollinated and a small portion of a leaf was painted with the herbicide Basta 7 days postpollination to identify transgene‐containing plants. If the transgenic‐encoded AGPase small subunit functioned in the endosperm to complement the mutant phenotype and in the absence of linkage to the endogenous *Bt2* gene, kernels on Basta‐resistant plants should exhibit a 15 wildtype to 1 mutant ratio. Events conditioning Basta resistance in the leaf and 15 to 1 wildtype to mutant kernel ratios (Figure S1) in self‐pollinated progeny were selected. These were then tested for cosegregation in the absence of the transgene (herbicide susceptibility) with the lack of a functional *Bt2* allele. These two tests identified two events harboring the wildtype gene construct, two events containing the MP mosaic gene and five events having the MP with QTCL motif (subsequently termed QTCL). These were then analyzed for alterations in maize yield. Transgenic events meeting these two criteria represented approximately 50% of the initial events.

Nonmutant seed from individual F_2_ progenies from the self‐pollination of plants hemizygous for the transgene (T/‐) and heterozygous at the *Bt2* locus (*Bt2/bt2*) above was planted, resulting plants were self‐pollinated, and a small section of the leaf was painted with Basta 7 days postpollination to identify transgene‐containing plants. Plants hemizygous for the transgene (Basta‐resistant) were *Bt2/Bt2*,* Bt2/bt2,* or *bt2/bt2* at the *bt2* locus. Plants of the first genotype did not segregate mutant seed on the selfed ear while those of the second and third genotypes produced 15 to 1 or 3 to 1 plump to mutant ratios, respectively, in the selfed generation. Plants homozygous for the transgene did not produce mutant seed in the selfed generation, and the genotype at the *bt2* locus could not be ascertained. Plants lacking the transgene (Basta‐sensitive) were either *Bt2/Bt2* or Bt2/bt2 at the *bt2* locus and inspection of selfed ear distinguished the two genotypes. The only plants not fully genotyped were Basta‐resistant not segregating mutant seed. These were hemizygous for the transgene and homozygous for *Bt2* or homozygous for the transgene.

Wildtype and mutant seed number per ear and individual seed weight were determined. These parameters were subsequently measured for seeds derived from self‐pollination of the F_3_ plants as well as plants from crosses to the inbred B73. In some cases, seeds from several sib F_3_ plants were pooled to generate the seeds planted in an individual experiment. Tests were performed over a three‐year period with multiple plantings throughout the season in some cases. Each experiment involved the planting of at least 45 seeds. The number and weight of mutant and nonmutant seeds per ear and the source of the seed planted are presented in Table S1. A ratio of each of these parameters is also presented in which the value generated from transgenic plants is divided by the value generated from sibling plants lacking the transgene in that particular experiment. Values greater than 1 then reflect an increase attributable to the transgene. Averages of these ratios for each transgenic event were calculated and are presented in Table 1. Perusal of Table 1 reveals that seed number, rather than seed weight, is increased by the presence of the transgene containing a modified AGPase small subunit. For example, averaged over five separate field experiments and environments, plants containing the MP‐2 transgenic event produced ears containing 57% more kernels than did sibling plants in each experiment that lacked the transgene.

**Table 1:** MP‐containing AGPase small subunit genes increase seed number in maize. Three versions of the AGPase maize endosperm small subunit gene, such as wildtype, MP, and MP containing the QTCL motif, were inserted into maize, and alterations in seed number and individual seed weight were monitored in separate events of each gene. Data given in the fourth, fifth, and sixth columns are the ratios in the various parameters of plants containing the transgene divided by plants lacking the transgene for seed number, individual plump seed weight, and individual mutant weight, respectively. Comparisons were made between sibling plants containing or lacking the transgene within the same population grown in the same environment. Experiments were replicated variable times. Differences in seed number significant at the 5% (*) and 1% (**) level are marked

Construct	Transgenic event	Number of replicates	Total seed number^a^	Plump seed weight	Mutant seed weight
MP	2	5	1.57**	0.91	0.98
MP	18	6	1.00	1.03	0.97
MP‐QTCL	42	3	1.21	1.04	1.26
MP‐QTCL	20	7	1.11	1.03	1.39
MP‐QTCL	24	1	1.43*	0.80	0.89
MP‐QTCL	35	1	1.62**	0.92	0.76
MP‐QTCL	44	1	1.16	1.00	1.05
Wildtype	57	4	0.92	1.03	1.00
Wildtype	51	2	0.93	0.92	0.65
Average			1.22	0.96	1.00
Average MP‐containing transgenes			1.30*	0.96	1.04
Average WT transgenes			0.93	0.98	0.83

a 1.35 without event MP‐18.

While the two constructs containing the wildtype *Bt2* gene and one of the MP transgenic events had no effect, the other MP event and all five of the MP‐QTCL constructs tested exhibited an average increase in seed number of 35%. Averaged for the various replications and excluding the MP‐18 construct, the MP‐containing constructs exhibited a seed number increase that is significant (*t* test) at the 0.38% level. Weights of individual wildtype seed and of mutant seed were 4% less and 4% greater than from plants lacking the transgene, respectively.

### 3.2 The MP‐containing transgenes increase yield by functioning in maternal tissue

Interestingly, the MP‐containing transgenes function in the plant to increase seed number. This conclusion can be gleaned from the fact that the presence of the transgene in the maternal parent increases the number of seeds in its self‐derived progeny, regardless of the seed genotype. Some F_3_ progeny produced from F_2_ plants that were hemizygous for the transgene and heterozygous at the *Bt2* locus contain seed that (i) are hemizygous for the transgene and lack a functional *Bt2* allele (*bt2/bt2*) and (ii) lack the transgene and are heterozygous at the *Bt2* (*Bt2/bt2*) locus. Plants derived from these two seed classes produce selfed progeny segregating 3 plump to 1 mutant ratios and the two classes can be distinguished by the herbicide resistance conditioned by the transgene.

Seed number and seed weight of plants of the two genotypes from each of the five events conditioning an increase in seed number are given in Table 2. While the transgene conditions an increase in the number of wildtype seed, it also increases the number of mutant seed even though the mutant seed does not contain the transgene. As an example, while event QTCL‐24 conditions an 84% increase in plump seed number (241/130.8), it also increases mutant seed 64% (75.67/46.25) even though the mutant seeds lack the transgene. Hence, while the MP‐containing transgene functions in the endosperm—as evidenced by the rescue of the mutant phenotype—its role in increasing seed number occurs in maternal tissue. These results are identical to the findings with a transgene for the endosperm AGPase large subunit (Hannah et al., 2012) which also functioned in maternal tissue to enhance seed number.

**Table 2:** MP‐containing AGPase small subunit genes function in maternal tissue to increase seed number. The number and weight (wt) of plump and mutant seed were determined in sibling progeny derived by self‐pollination of plants with the genotypes MP/‐; bt2/bt2 and no MP/Bt2/bt2. Differences in seed number significant at the 5% (*) and 1% (**) level are marked

Event	Parental genotype	Average seed #		Average seed wt.	
		Plp	Mut	Plp	Mut
MP‐2	MP/‐; *bt2/bt2*	276	71	0.2	0.10
	No MP; *Bt2/bt2*	78	17	0.20	0.11
	Ratio	3.52**	4.19**	0.97	0.90
QTCL‐24	MP/‐; *bt2/bt2*	241.	75.667	0.18	0.09
	No MP; *Bt2/bt2*	131	46	0.23	0.11
	Ratio	1.84**	1.64*	0.78	0.78
QTCL‐35	MP/‐; *bt2/bt2*	258	78	0.20	0.11
	No MP; *Bt2/bt2*	201	44	0.23	0.16
	Ratio	1.28	1.76	0.87	0.70
QTCL‐44	MP/‐; *bt2/bt2*	252	76.57	0.22	0.12
	No MP; *Bt2/bt2*	233	75	0.22	0.10
	Ratio	1.08	1.03	0.97	1.23
QTCL‐20	MP/‐; *bt2/bt2*	421	92	0.180	0.09
	No MP; *Bt2/bt2*	268	90	0.22	0.07
	Ratio	1.57*	1.02	0.81	1.32
Average		1.86	1.93	0.88	0.99

An interesting characteristic of the *HS33/Rev6 Sh2* AGPase large subunit transgene noted by Hannah et al. (2012) was a positive correlation between the extent of increase in seed number and the temperature during the first 4 days following pollination. Daily high temperatures above 33°C were associated with greater transgene‐conditioned increases in seed number. Seed number increase however was not found below 33°C.

Seed number increase and average daily high temperature during the first 4 days post pollination are shown in Table 3 for each experiment of transgenic events conditioning an increase in seed number. Within each transgene, greater increases in seed number were routinely associated with higher temperature. Of the 18 experiments, six yielded seed number ratios of 1.00 or less. Five of the six occurred when the average daily high temperature during the first 4 days postpollination was below 33°C. A correlation of 0.22 exists between seed increase and temperature across all transgenes. It is interesting to note that wildtype constructs and MP‐18 were sampled under high‐temperature regimes and no increase in seed number was observed.

**Table 3:** Seed number increase is sensitive to temperature at the time of seed set. The average increase in total seed number caused by the transgene and the average daily high temperature during the first 4 days following pollination are given for each experiment for the six MP‐containing transgenic events that increase seed number. Differences in seed number significant at the 5% (*) and 1% (**) level are marked

Gene	Event	Season	Avg seed # increase	Avg high temp 0–4 dpp
MP	2	Spring 07	2.67**	32.71
MP	2	Spring 08	2.22*	31.74
MP	2	Spring 09	1.64*	33.83
MP	2	Spring 09	1.23	30.00
MP	2	Spring 09	0.92	30.99
QTCL in MP	20	Spring 08	1.56**	35.57
QTCL in MP	20	Spring 07	1.40**	33.93
QTCL in MP	20	Spring 08	1.30*	31.74
QTCL in MP	20	Spring 08	1.00	31.31
QTCL in MP	20	Spring 09	1.00	29.24
QTCL in MP	20	Spring 09	0.84	32.73
QTCL in MP	20	Spring 09	0.64	34.09
QTCL in MP	24	Spring 07	1.43*	34.37
QTCL in MP	35	Spring 07	1.62**	33.66
QTCL in MP	42	Spring 08	1.59**	35.57
QTCL in MP	42	Spring 08	1.08	32.18
QTCL in MP	42	Spring 07	0.96	31.81
QTCL in MP	44	Spring 07	1.16	33.88

To summarize, MP‐containing AGPase small subunit genes, like the *HS33/Rev6 Sh2* AGPase large subunit transgene reported earlier (i) function in the endosperm to produce a wildtype seed phenotype, (ii) increase seed number by functioning in maternal tissue, (iii) exhibit greater increases in seed number when daily high temperatures during the first 4 days following pollination average 33°C or higher but (iv) do not increase individual seed weight.

### 3.3 The MP AGPase exhibits altered enzyme properties in both *E. coli* and the maize endosperm

To investigate the lack of increase in seed weight, we first asked whether the altered enzymological properties exhibited by the MP‐encoded AGPase in *E. coli* also occurred when the enzyme was expressed in the maize endosperm. Accordingly, we developed sibling stocks in which one population expressed only MP as the active AGPase small subunit while the sibling stock expressed only the endogenous *Bt2‐*encoded AGPase small subunit. Progeny from self‐pollination of a plant hemizygous for the MP‐*2* insertional event and heterozygous at the *bt2* locus (*Bt2/bt2*) yielded progeny of the genotypes (a) *MP‐2/MP‐2;bt2/bt2* and (b) −/−; *Bt2/Bt2*. The first genotype was identified through molecular markers for the *bt2* alleles, and by the true breeding nature of plump seed and herbicide‐resistant plants. The second genotype did not segregate mutant seed in subsequent generations and did not produce herbicide‐resistant plants.

**Figure 1:**
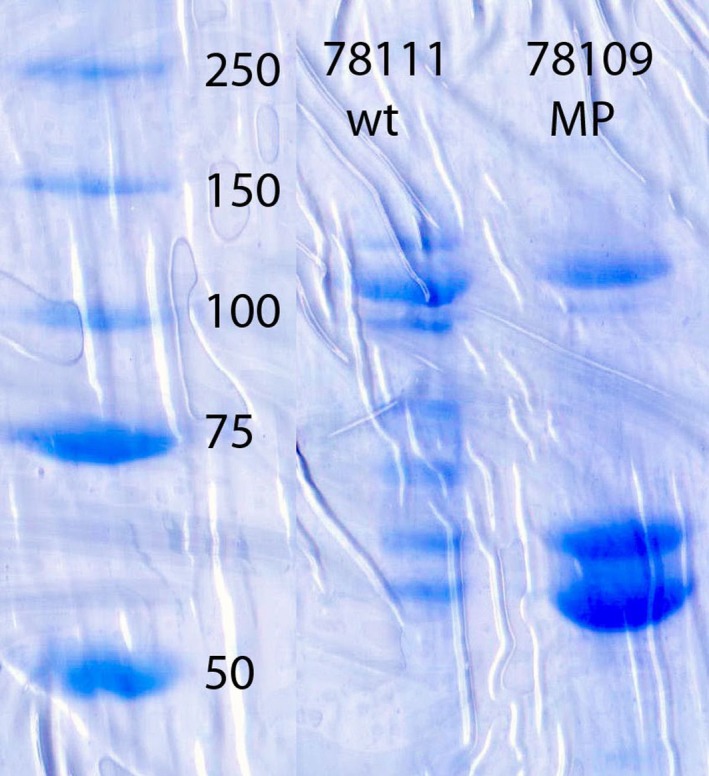
SDS polyacrylamide gel of highly purified ADP‐glucose pyrophosphorylase from 18‐day‐old endosperms expressing either the *Bt2* or *MP* small subunit. Calculated molecular weights of the small and large subunit of ADP‐glucose pyrophosphorylase are 52 kD and 57 kD. Molecular weight markers are shown in the first lane

AGPase from 18‐day‐old kernels of these two genotypes was harvested and AGPase was purified as described (Section 2.2 and Boehlein et al., 2005). Reducing SDS gels of the two preparations are shown in Figure 1. The two proteins with molecular weights of 52 kD and 57 kD are the small and large subunits of AGPase. Because of enhanced stability encoded by MP, a larger proportion of the enzyme remains intact during purification, giving rise to greater fold purification of MP preparations relative to wildtype.

The catalytic stability as a function of temperature was determined for both kernel preparations. MP and wildtype AGPase purified from our *E. coli* expression system were also included as controls. Previously, we (Boehlein, Shaw, Stewart, & Hannah, 2010; Boehlein, Shaw, McCarty et al., 2013) showed that *E. coli‐*expressed MP AGPase is quite active at 55°C and exhibited activity at this elevated temperature that was comparable to that seen at 37°C. In contrast, the *E. coli‐*expressed wildtype enzyme exhibited very little activity at 55°C. Enzyme reaction mixtures were incubated at the two temperatures, and activity rates were measured for the four enzyme preparations (Table 4). The pattern seen with the *E. coli‐*expressed enzymes is also seen with the endosperm‐expressed enzymes. Regardless of the host, activity of the MP AGPase is comparable at both 37°C and 55°C, whereas the wildtype enzyme exhibits very little or no activity at 55°C.

**Table 4:** Catalytic stability of ADP‐glucose pyrophosphorylases encoded by *MPSh2* and *Bt2Sh2* in the maize endosperm and in *Escherichia coli*. Activity is reported in μmol/min/mg

Genotype	Host	Activity at 55°C	Activity at 37°C
*MPSh2*	Endosperm	13.2 ± 0.86	9.8 ± 0.16
*MPSh2*	*E. coli*	23.2 ± 1.8	21.9 ± 1.5
*Bt2Sh2*	Endosperm	0	2.5 ± 0.12
*Bt2Sh2*	*E. coli*	1.2 ± 0.21	11.8 ± 0.79

The thermodynamic stability of the four enzyme preparations was also measured. Previous work from our laboratory has shown that the binding of AGPase to a reactant, activator or inhibitor, as occurs during catalysis, greatly stabilizes the enzyme to heat‐induced activity loss. Hence, the stability of the four enzyme preparations was measured in the absence of any known molecule that binds to the enzyme. Desalted AGPase in a 50 mM HEPES pH 7.4 containing 1 mg/ml BSA was incubated in water baths at 37°C, 42°C or 55°C for various times and the *t*_½_ was determined (Table 5). The MP‐containing AGPase expressed in either the endosperm or in *E. coli* exhibited greater stability at each of the three temperatures compared to the wildtype enzyme expressed in either host.

**Table 5:** Thermodynamic stability of ADP‐glucose pyrophosphorylases encoded by *MPSh2* and *Bt2Sh2* in the maize endosperm and in *Escherichia coli*. *T*_½_ values are given in minutes

Genotype	Host	*T*_½_ at 37°C	*T*_½_ at 42°C	*T*_½_ at 55°C
*MPSh2*	Endosperm	68	18.4	1.6
*MPSh2*	*E. coli*	49	9.2	0.8
*Bt2Sh2*	Endosperm	23	5.8	<0.5
*Bt2Sh2*	*E. coli*	10	3.3	<0.5

A significant feature of the MP‐encoded AGPase from *E. coli* is the magnitude of activity in the absence of the activator 3PGA. Accordingly, we examined the endosperm‐encoded MP enzyme for 3PGA‐independent activity (Table 6). The vast majority of both the endosperm‐encoded and the *E. coli*‐encoded MP AGPase activity was 3PGA‐independent, whereas the wildtype enzyme from either host was virtually dependent on 3PGA for activity.

**Table 6:** 3‐Phosphoglyceric acid activation of ADP‐glucose pyrophosphorylases encoded by *MPSh2* and *Bt2Sh2* in the maize endosperm and in *Escherichia coli*

Genotype	Host	*K*_*a*_ (mM)	*V*_min_ a	*V* b	% Activity – 3PGA^c^
*MPSh2*	Endosperm	0.082 ± 0.029	6.70 ± 0.17	2.05 ± 0.24	77
*MPSh2*	*E. coli*	0.060 ± 0.046	21.43 ± 1.00	5.65 ± 1.37	79
*Bt2Sh2*	Endosperm	0.081 ± 0.007	—	2.74 ± 0.07	0
*Bt2Sh2*	*E. coli*	0.049 ± 0.009	1.78 ± 0.49	10.78 ± 0.61	15

a *V*_min_ is activity in the absence of 3PGA.

b *V* is the increase in activity caused by 3PGA.

c Is the percentage of total activity observed in the absence of 3PGA. Activity is reported in μmol/min/mg.

While allosteric and heat stability properties of the *E. coli*‐expressed MP enzyme were significantly altered, kinetic parameters were unaffected. These parameters were monitored in the endosperm‐expressed enzymes (Table 7). *K*_*m*_ values for the physiological substrates, glucose‐1‐phosphate, and ATP were comparable for all four enzyme preparations.

**Table 7:** Michaelis constants for ATP and glucose‐1‐phosphate of ADP‐glucose pyrophosphorylases encoded by *MPSh2* and *Bt2Sh2* in the maize endosperm and in *Escherichia coli*

Genotype	Host	*K*_*m*_ ATP (mM)	*K*_*m*_ G‐1‐P (mM)	*V*_max_ (nmol/min/mg)
*MPSh2*	Endosperm	0.059 ± 0.006	0.041 ± 0.008	10,200 ± 300
*MPSh2*	*E. coli*	0.056 ± 0.010	0.039 ± 0.004	15,100 ± 450
*Bt2Sh2*	Endosperm	0.044 ± 0.005	0.042 ± 0.002	2,800 ± 35
*Bt2Sh2*	*E. coli*	0.057 ± 0.004	0.030 ± 0.006	9,400 ± 200

In summary, the altered enzyme properties of MP noted in *E. coli* are also found in the maize expressed AGPase.

## 4 DISCUSSION

Previously, we (Hannah et al., 2012) engineered the *Sh2*‐encoded large subunit of the endosperm AGPase for enhanced heat stability and reduced sensitivity to an inhibitor and placed the gene into maize. Unexpectedly, we found that while the transgene was expressed in the endosperm and could rescue or complement the mutant endosperm phenotype, the transgene increased yield by functioning in the plant to enhance the probability that an ovary developed into a functional kernel. The phenomenon was found to be temperature‐dependent and occurred only when high temperatures during the first 4 days following pollination averaged 33°C or higher. While transcripts of the transgene could be found in multiple tissues, the AGPase having the product of the transgene could only be found in the endosperm.

Here, we show that an engineered small subunit of the endosperm AGPase targeted for expression in the endosperm exhibits the same phenotypic changes as noted with the engineered large subunit: While it is operational in the endosperm, it also functions in the plant to increase seed number and the extent of seed number increase is dependent on the high temperature during the first 4 days following pollination.

The mechanism by which these presumed endosperm‐specific genes function in a plant tissue is not obvious. Starch synthesis in the endosperm is fundamentally different than synthesis in other tissues as the AGPase is cytosolic in the endosperm but thought to be plastid localized in all other tissues (reviewed in Smith et al., 1997; Ballicora et al., 2003; Hannah, 2007; Hannah & Greene, 2008; Hannah & James, 2008; Preiss, 2009; Keeling & Myers, 2010). The endosperm amyloplast contains an ADP‐glucose transporter encoded by the *brittle‐1* gene that appears only to be expressed in the endosperm (http://qteller.com/). Perhaps endosperm‐type starch synthesis occurs only transiently in a plant tissue and has yet to be detected.

The altered heat stability and allosteric properties introduced into the BT2 protein appear important in increasing seed number as incorporation of the wildtype *Bt2* gene did not increase seed number. Allosterically enhanced AGPases have been created and exploited following the classic work with the *glgC‐16* mutant in *E. coli* (Leung et al., 1986). This mutation accumulates glycogen more quickly than does wildtype *E. coli* and conditions an AGPase with less sensitivity to the inhibitor 5′‐AMP and less dependence on the activator fructose 1,6 bis‐phosphate. Placement of this variant into potato increases tuber starch yield 35% (Stark et al., 1992); however, incorporation of the wildtype *E. coli* gene into potato had no effect. Heat stability of AGPase was targeted for engineering following a whole series of maize studies (Keeling, Banisadr, Barone, Wasserman, & Singletary, 1994; Singletary, Banisadr, & Keeling, 1993, 1994; Wilhelm, Mullen, Keeling, & Singletary, 1999), showing that high temperatures during kernel development decreased yield and endosperm AGPase. Of the various enzymes assayed, only AGPase and starch synthase activities were reduced in the high‐temperature environment.

Interestingly, while the altered allosteric and/or heat stability properties of the incorporated AGPase small subunit appear important in increasing seed number, these alterations did not increase individual seed weight. Perhaps the altered allosteric and heat stability properties are not important in increasing starch synthesis in the maize endosperm. Alternatively, given the inverse relationship normally seen between maize seed number and individual seed weight (reviewed in Gambin & Borras, 2010), the lack of a seed weight reduction concomitant with the enhanced seed number may be attributable to the enhanced allosteric and heat stability properties of the inserted AGPase.

## AUTHOR CONTRIBUTION

LCH supervised the project and wrote the article, JRS genotyped the plants and generated seed number, MAC synthesized the vectors for transformation and SKB performed the enzymatic work. NG provided technical assistance. JRS, MAC, NG, and SKB performed the experiments.
