# Supplementary material for: A brittle‐2 transgene increases maize yield by acting in maternal tissues to increase seed number
Source: Plant Direct. 2017 Dec 7;1(6):e00029. doi: 10.1002/pld3.29 (PMC6508519; doi:10.1002/pld3.29)
Supplement: Supplementary file 1 [file PLD3-1-e00029-s001.pdf]

Figure S1. Crossing scheme for studies of the transgenes (T)

Original Cross: -/-;*bt2/bt2* X T/-; *Bt2/Bt2* ⟶ T/-; *Bt2/bt2* & -/- *Bt2/bt2*

Transgene containing plants from cross above selected by Basta resistance

Self pollination of T/-;*Bt2/bt2* plants yields the following genotypes:

(1)	T/ ;*Bt2/*	9
(2)	T/ ;*bt2/bt2*	3
(3)	-/- ;*Bt2/*	3
(4)	-/-; *bt2/bt2*	1

If the transgene functions in the endosperm to complement the *bt2* mutant, then seeds of genotype (2) are wildtype (plump) and a 15 plump to 1 mutant ratio is produced. If no complementation, then a 3 plump to 1 mutant ratio is produced.
