# Supplementary material for: A brittle‐2 transgene increases maize yield by acting in maternal tissues to increase seed number
Source: Plant Direct. 2017 Dec 7;1(6):e00029. doi: 10.1002/pld3.29 (PMC6508519; doi:10.1002/pld3.29)
Supplement: Supplementary file 2 [file PLD3-1-e00029-s002.pdf]

Table S1.

Gene	Event	Row	Trans-gene	Temp	# ears	total seed	wt/ plump	wt/ shrunk
**WT**	**51**	66218	yes	31.81	3	120.00	0.19	
		F2	**ratio**			**0.77**	**0.92**	
			No		4	156.75	0.21	
WT	51	64042	yes	33.44	11	278.80	0.21	0.11
		F2	**ratio**			**1.10**	**0.92**	**0.67**
			No		19	276.89	0.24	0.17
WT	57	64051	yes	32.59	26	321.85	0.22	0.09
		F2	**ratio**			**0.87**	**0.96**	**0.86**
			No		9	369.56	0.23	0.11
WT	57	66348	yes	32.33	17	342.47	0.26	0.09
		Cross to B73	**ratio**			**0.94**	**1.07**	**1.12**
			No		4	365.75	0.25	0.08
WT	57	66448	yes	31.45	9	225.78	0.24	0.08
		Cross to B73	**ratio**			**1.03**	**1.05**	**0.91**
			No		5	220.00	0.23	0.09
**WT**	**57**	66222	yes	35.39	8	336.50	0.31	0.23
		Cross to B73	**ratio**			**0.86**	**1.04**	**1.13**
			No		10	389.20	0.29	0.20
**MP**	**2**	64021	yes	32.71	28	364.57	0.20	0.08
		F2	**ratio**			**2.67****	**0.94**	**0.75**
			No		6	136.33	0.21	0.11
**MP**	**2**	68095	yes	30.99	44	258.00	0.15	0.12
F3 from sib F2 plants			**ratio**			**0.92**	**0.77**	**1.41**
			No		7	279.50	0.19	0.08
**MP**	**2**	68226	yes	30.00	24	354.75	0.19	0.10
F3 from sib F2 plants			**ratio**			**1.23**	**0.93**	**0.92**
			No		8	288.33	0.20	0.10
**MP**	**2**	68364	yes	33.83	16	312.63	0.18	0.08
F3 from sib F2 plants			**ratio**			**1.64***	**0.83**	**0.69**
			No		5	190.40	0.22	0.12
MP	2	66332	yes	31.74	3	380.00	0.25	0.07
		Cross to B73	**ratio**			**2.21***	**1.09**	**1.15**
			No		3	171.33	0.23	0.06
**MP**	**18**	66209	yes	34.90	11	418.82	0.30	0.11
		Cross to B73	**ratio**			**0.99**	**1.08**	**1.05**
			No		17	421.00	0.27	0.11
**MP**	**18**	66336	yes	32.03	7	441.29	0.27	0.08
		Cross to B73	**ratio**			**1.23**	**1.01**	**0.89**
			No		14	359.50	0.27	0.09

**MP**	**18**	66436	yes	31.34	8	308.50	0.24	0.09
		Cross to B73	**ratio**			**1.16**	**1.03**	**0.93**
			No		10	265.10	0.23	0.09
**MP**	**18**	68107	yes	30.80	33	330.12	0.23	0.09
F3 from sib F2 plants			**ratio**			**0.91**	**1.10**	**1.09**
			No		6	364.67	0.21	0.09
**MP**	**18**	68238	yes	33.11	22	275.32	0.20	0.10
F3 from sib F2 plants			**ratio**			**0.81**	**0.95**	**1.06**
			No		3	340.33	0.21	0.09
**MP**	**18**	68376	yes	33.86	24	283.25	0.23	0.09
F3 from sib F2 plants			**ratio**			**0.88**	**0.99**	**0.77**
			No		4	322.25	0.23	0.11
**QTCL in MI**	**42**	64039	yes	31.81	5	280.79	0.23	0.11
		F2	**ratio**			**0.96**	**1.12**	
			No		2	291.00	0.20	
**QTCL in MI**	**42**	66215	yes	35.57	4	485.50	0.26	0.10
		Cross to B73	**ratio**			**1.59****	**1.02**	**0.94**
			No		10	304.60	0.25	0.11
QTCL in MP	42	66342	yes	32.18	7	460.33	0.25	0.16
		Cross to B73	**ratio**			**1.08**	**0.98**	**1.58**
			No		5	426.80	0.25	0.10
QTCL in MP	20	64027	yes	33.93	17	424.82	0.18	0.25
		F2	**ratio**			1.40**	0.83	3.47
			No		5	304.00	0.22	0.07
QTCL in MP	20	66211	yes	35.57	12	492.08	0.27	0.10
		Cross to B73	**ratio**			**1.56****	**0.96**	**0.90**
			No		13	314.54	0.28	0.12
QTCL in MP	20	66338	yes	31.74	9	446.50	0.25	0.11
		Cross to B73	**ratio**			**1.29***	**1.04**	**1.17**
			No		9	344.22	0.24	0.09
QTCL in MP	20	66437	yes	31.31	13	288.15	0.24	0.10
		Cross to B73	**ratio**			**1.00**	**1.14**	**1.17**
			No		12	288.42	0.21	0.09
QTCL in MP	20	68081	yes	29.24	42	256.21	0.21	0.10
F3 from sib F2 plants			**ratio**			**1.00**	**0.95**	**0.91**
			No		12	291.75	0.22	0.11
QTCL in MP	20	68212	yes	32.73	11	306.18	0.20	0.09
F3 from sib F2 plants			**ratio**			**0.84**	**1.27**	**1.39**
			No		6	365.67	0.16	0.07
QTCL in MP	20	68350	yes	34.09	9	207.20	0.19	0.06
F3 from sib F2 plants			**ratio**			**0.64**	**1.00**	**0.71**
			No		2	321.50	0.19	0.09
QTCL in MP	24	64030	yes	34.37	14	231.57	0.18	0.11
		F2	**ratio**			**1.43***	**0.79**	
			No		6	161.67	0.23	
QTCL in MP	35	64036	yes	33.66	13	373.13	0.22	0.13
		F2	**ratio**			**1.62****	**0.92**	

			No		6	230.83	0.23	
QTCL in MP	44	64048	yes	33.88	26	334.88	0.22	0.11
		F2	**ratio**			**1.16**	**1.01**	**1.04**
			No		8	289.13	0.22	0.10
